# Multi-FusNet–convolutional neural network with improved Huber loss function for plant leaf disease detection and classification

**DOI:** 10.3389/fpls.2026.1787185

**Published:** 2026-05-04

**Authors:** B. S. Shruthi, M. S. Narasimha Murthy, Eman Abdullah Aldakheel, Doaa Sami Khafaga

**Affiliations:** 1 Department of Computer Science and Engineering, Malnad College of Engineering, Hassan, India, Belagavi, India; 2 Department of Computer Science and Engineering, BMS Institute of Technology and Management, Bangalore, India, Belagavi, India; 3 Department of Computer Sciences, College of Computer and Information Sciences, Princess Nourah bint Abdulrahman University, Riyadh, Saudi Arabia

**Keywords:** convolutional neural network, generalization capability, Huber loss, plant disease detection, residual network

## Abstract

**Background:**

Recently, plant disease detection and classification have become major concerns in agriculture. Early detection of plant diseases supports farmers to take precautionary actions to prevent the spread of infections across different parts of the plant. However, detecting and classifying plant leaf diseases remain challenging tasks due to the overlapping characteristics of different diseases.

**Methods:**

To mitigate these limitations, this research developed a Multi-FusNet–convolutional neural network (Multi-FusNet–CNN) with an improved Huber loss function to classify multiple classes of plant leaf diseases. Here, a multipath residual network (Multi-RG) with cross-filtering fusion is integrated, and the pixel shuffling fusion method is developed for fusing low-level to up-sampled features. An improved Huber loss function is incorporated into the Multi-FusNet–CNN to effectively handle outliers and enhance the model’s generalization capability during training.

**Results:**

The developed Multi-FusNet–CNN with improved Huber loss function achieved 99.95% accuracy, 99.13% F1-score, 99.87% recall, 99.27% precision, and 99.93% specificity, thereby outperforming existing conventional techniques.

**Conclusion:**

The proposed Multi-FusNet–CNN model improved the generalization capability of the method during the training process on plant leaf disease detection and classification.

## 1 Introduction

Plant diseases have a significant impact on crop health, causing extensive damage and reducing yields,while also resulting in substantial financial losses for farmers (Falaschetti et al., 2022; Moupojou et al., 2023; Tabbakh and Barpanda, 2023). The detection and classification of plant leaf diseases play an essential role in modern horticulture and agriculture, ensuring crop productivity and resilience (Ali et al., 2024; Sinanc Terzi, 2024). These diseases include various pathogens, infections, and abnormalities that affect plant growth and overall health (Ndovie and Masabo, 2024). They are caused by fungi, bacteria, nematodes, viruses, or environmental factors such as nutrient deficiencies and adverse weather conditions (Chen et al., 2023). Accurate detection of plant diseases plays a crucial role in developing efficient disease management and protection strategies (Albattah et al., 2022; Ashwini and Sellam, 2023; Paramanandham et al., 2024). Traditional plant disease detection algorithms primarily rely on manual visual evaluation by experts (Pandey and Jain, 2022). However, these techniques have several limitations, including time-consuming, prone to human error, and subject to variability (Pal and Kumar, 2023). Recently, automatic plant disease detection systems have been developed to effectively overcome the drawbacks of conventional techniques (Kurmi and Gangwar, 2022).

In recent years, researchers have developed deep learning (DL)-based algorithms that utilize high-performance devices equipped with servers and graphics processing units (GPUs) (Hasan S. et al., 2022). However, the utilization of sophisticated GPU-equipped devices is not practical in agriculture settings due to their high cost (Prabu and Chelliah, 2022; Singh et al., 2022; Duhan et al., 2024; Hemalatha and Jayachandran, 2024). Hence, there is a requirement for applications with fewer parameters, lower energy consumption, and efficient execution (Sai Reddy and Neeraja, 2022). Convolutional neural networks (CNNs) have been widely used for plant disease detection because of their ability to capture spatial dependencies in images (Salam et al., 2024). These networks are developed with multiple layers of convolutional and pooling processes, allowing them to learn hierarchical representations of input images (Balafas et al., 2023; Shrotriya et al., 2024). CNNs have demonstrated effective performance in various plant disease detection tasks, thereby achieving high robustness and accuracy (Ahamed et al., 2024; Aboelenin et al., 2025).

### 1.1 Problem statement

In the agricultural sector, plant diseases have a critical impact on crop health, resulting in reduced yields and significant financial losses for farmers. Traditional disease detection algorithms, which are primarily based on manual visual inspection, arelabor-intensive, prone to errors, and not scalable for large-scale farming. Additionally, the detection and classification of plant leaf diseases are challenging because of overlapping disease symptoms and variability in environmental conditions. Existing DL-based algorithms often require high computational resources and are sensitive to outliers, which limits their practical deployment in agricultural environments.

### 1.2 Objective

The primary aim of this research is to develop a novel Multi-FusNet–CNN model combined with an improved Huber loss function for plant leaf disease detection and classification. The proposed method aims to mitigate the challenges posed by overlapping disease symptoms and enhance the generalization ability of traditional CNNs by incorporating multipath residual architectures, cross-filtering fusion, and pixel shuffle fusion techniques. By reducing the sensitivity to outliers and minimizing computational complexity, the developed algorithm achieves high accuracy, thereby making it suitable for real-world agricultural applications.

### 1.3 Contributions

The primary contributions of this research are summarized as follows:

- Median filter and Min–Max normalization techniques are employed in the pre-processing phase, which remove noise and normalize the pixel intensity values in the images.
- The Multi-FusNet–convolutional neural network (Multi-FusNet–CNN) with improved Huber loss function is developed in the classification phase, which includes residual blocks and pixel shuffle Net to classify different categories of plant diseases.
- The improved Huber loss function is incorporated in the Multi-FusNet algorithm, which helps stabilize the training process and enhance the model’s generalization capability.

This research paper is organized as follows: Section 2 summarizes existing research. Section 3 describes the process of the proposed methodology. Section 4 explains the outcomes and discussion of the developed approach. Finally, Section 5 concludes the paper.

## 2 Literature review

In this section, existing algorithms are analyzed as ML-based approaches and CNN-based approaches with their advantages and disadvantages.

### 2.1 ML-based approaches

Kotwal et al. (2024) suggested a hybrid strategy based on an optimized automatic system for Plant Leaf Disease Classification (PLDC). First, the suggested method performed pre-processing by utilizing Gaussian filtering and image resizing. Next, disease-infected areas were segmented by the UNet method to obtain relevant areas and improve disease classification accuracy. The weights of the UNet method were tuned by applying the hunter–prey optimization (Hunt-PO) algorithm. Feature extraction was then performed usinggray-level co-occurrence matrix (GLCM), scale-invariant feature transform (SIFT), and Gabor filter to extract essential features for classification. Based on the extracted features, the suggested method was implemented by artificial driving-EfficientNet (AD-ENet). Sahu and Pandey (2023) presented a hybrid random forest–multiclass support vector machine (HRF-MCSVM) model for disease detection. To enhance execution accuracy, image features were segmented by spatial fuzzy C-means for the classification process. Reddy and Adimoolam (2022) developed the K-nearestneighbor (K-NN) algorithm for plant disease detection and compared its accuracy with the naïve Bayes (NB) technique. In the developed method, the detection of plant leaf disease was performed using ML algorithms like KNN (*N* = 10) and NB (*N* = 10), and their accuracies were evaluated for similar classes. Panigrahi et al. (2020) introduced supervised ML algorithms like NB, decision tree (DT), K-NN, SVM, and random forest (RF) to detect maize plant disease. These algorithms were analyzed and compared to determine the most suitable method with the highest accuracy for plant disease prediction. Gosai et al. (2022) implemented the residual neural network (ResNet), a subphase of artificial neural network (ANN). The ResNet technique included residual block, which was utilized to resolve the issue of exploding or vanishing gradients. The ResNet algorithm was also used for developing the residual network and achieved promising results. The implemented method employed certain parameters such as gradient clipping, scheduling learning rate, and weight decay.

From the analysis of the above ML-based algorithms, it is concluded that these traditional techniques were easy to utilize but required a large quantity of training data. These processes were not difficult for major changes in size, color, and types of plant leaf diseases occurring in the environment. Therefore, a more robust strategy utilizing many advanced techniques was required to enhance accuracy in identifying various plant leaf diseases.

### 2.2 CNN-based approaches

Hassan and Maji (2022) developed a novel DL-based algorithm that relied on an inception layer and residual connection. Depthwise separable convolution was utilized to minimize the number of parameters. The developed architecture utilized less parameters than other DL algorithms and was quicker than conventional DL algorithms. Moussafir et al. (2022) implemented a reliable tool for early disease diagnosis for farmers. Imaging was the essential method for diagnosing and quantifying disease plots. Feasible automatic and non-intrusive imaging was enabled with lower human resource and instrumentation costs, accounting for local agricultural priorities in large production regions. The primary aim of the implemented method was to develop a hybrid model for tomato disease detection based on image data collection. Transfer learning and fine-tuning schemes were employed to enhance the performance of various pre-trained techniques. Two CNN-based techniques were selected to develop the hybrid method for plant disease detection.

Reddy et al. (2023) suggested the customized PDICNet model for plant leaf disease detection and classification. First, the ResNet-50 was introduced to extract numerous attributes from plant leaf images, including texture and color characteristics. Additionally, the modified red deer optimization algorithm (MRDOA) was introduced as an optimal feature selection approach to obtain optimized and salient attributeswhile minimizing the model size. Then, a deep learning convolutional neural network (DLCNN) approach was used to achieve improved classification performance. Rizwan et al. (2024) introduced CNN-based prominent techniques such as Inception, MobileNet, ResNet50, and Xception for automatic plant disease detection due to their effective performance. However, these methods required substantial executional resources, thereby restricting their usage for large-scale farmers. The introduced method was more suitable for small-scale farmers. Ashwinkumar et al. (2022) presented an automated method for identifying and classifying plant leaf diseases by optimal mobile network-based CNN (OMNCNN). The presented method was processed on various stages such as pre-processing, segmentation, feature extraction, and classification. Bilateral filtering (BF) and Kapur’s thresholding techniques were used to identify damaged regions of leaf images. Moreover, the MobileNet technique was employed as the feature extraction model, and its hyperparameters were optimized through the usage of the emperor penguin optimizer (EPO) for improving the detection rate of plant diseases. Finally, an extreme learning machine (ELM)-based classifier was utilized to allocate suitable class labels for plant leaf images.

### 2.3 Coffee plant-based disease detection

Faisal et al. (2023) presented a hybrid feature fusion technique to identify coffee leaf diseases, including early and late feature fusion. Initially, several hybrid methods were developed to extract features from input images by integrating MobileNetV3, Swin Transformer, and variational autoencoder (VAE). MobileNet V3 acted as an inductive locality bias model, extracting nearby image attributes. These various extracted attributes included complementary data that enriched the unified feature map. Next, the extracted images from the method were integrated into an early fusion network. The late fusion method was then introduced for comprehensive learning of the fused features prior to the identification of coffee leaf diseases in the classification stage. Hasan RI. et al. (2022) suggested a framework to mitigate the issues of the conventional segmentation technique to generate masks for deep disease classification methods. The primary objective was to label datasets based on semi-automated segmentation of leaves and confused areas. As a result, each pixel on predetermined lesions was precisely selected.

#### 2.3.1 Model selection

In this research, a DL-based algorithm named Multi-FusNet–CNN is selected to address the challenges in plant leaf disease detection and classification. The selection of this method is motivated by the requirement for a lightweight yet highly accurate architecture, which is capable of handling overlapping disease symptoms, reducing computational cost, and enhancing generalization capability. Conventional CNN algorithms often suffer from overfitting, high parameter counts, and poor handling of outliers.

To mitigate these issues, the Multi-FusNet architecture combines a multipath residual network, cross-filtration fusion, and a pixel shuffle fusion mechanism for improving feature extraction and preservation of fine details. Moreover, the inclusion of an improved Huber loss function ensures robustness to outliers and smoothens the model optimization process compared with standard loss functions such as cross-entropy or MSE. This method is selected after a thorough analysis of existing algorithms, considering performance metrics and computational efficacy, making it highly suitable for real-world agricultural applications, where resources are often limited.

## 3 Dataset

**Figure 1:**
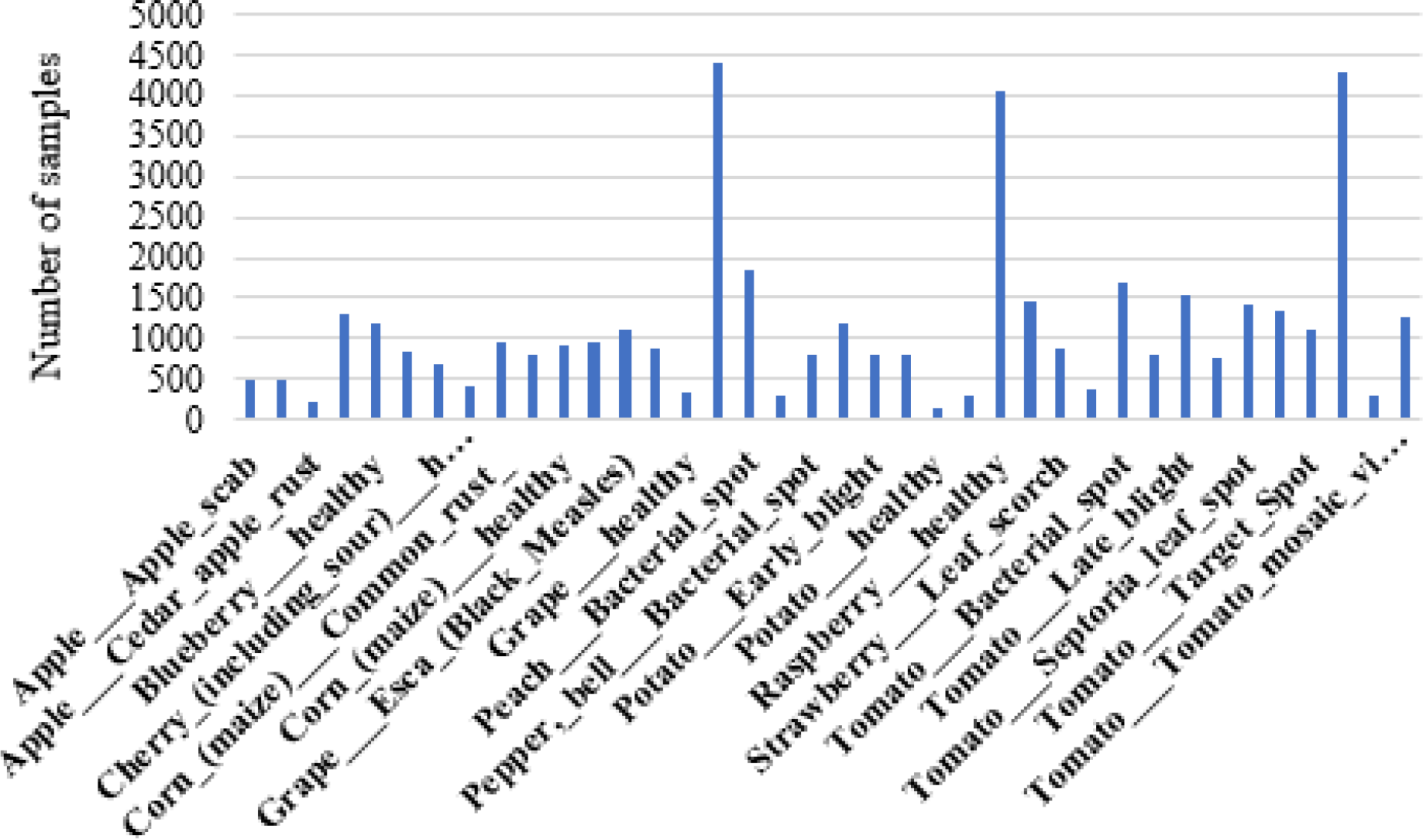
Class distribution of the plant village dataset.

A large quantity of data is required to efficiently train DL-based techniques. For effective training of models, the dataset must include certain visual features involving illumination and differences in shape, location, color, and leaf size. Considering these features, certain datasets are explored, including DiaMOS, Plant Village, PDD271 and PlantDoc. The PlantDoc and DiaMOS datasets contain a limited number of classes and samples, whereas the other two datasets comprise a large number of classes and samples, but the PDD271 dataset is not publicly available. The Plant Village dataset (Plant village dataset) consists of 54,305 samples distributed across 38 classes of plant diseases. In addition, it includes one extra class containing 1,143 background images, bringing the total number of images in the dataset to 55,448. **Figure 1** presents the class distribution of the Plant Village dataset.

### 3.1 RoCoLe dataset

This dataset includes 1,560 samples of Robusta coffee leaves (RoCoLe dataset), comprising both healthy and diseased instances (rust and red spider mite) at various phases. Each sample contains one symptom; the samples are considered in the field with various weather conditions and challenging backgrounds.

### 3.2 Coffee yield data

In this dataset, the biennial nature of coffee production is considered. The 2005 harvest is regarded as a high-yield year compared with the 2006 harvest. Hence, it is essential for establishing various thresholds to classify the training data of models for estimating yield classes for each year. Raster surfaces interpolated on a grid with a 30-m spatial resolution are transferred into spatial points with features for acquiring the reference of one yield point per pixel for each crop year. Subsequently, analysis of quantile criteria is utilized to classify the data into five classes. Different thresholds defined the yield classes due to similar number of points in every determined class threshold. **Table 1** describes the coffee yield dataset.

**Table 1:** Coffee yield dataset description.

Harvest	Quantile interval	Number of points	Class number
2005	0.550–12.138	21	1
2005	12.138–17.586	20	2
2005	17.586–22.348	20	3
2005	22.348–32.640	19	4
2005	32.640–59.350	22	5
2006	0.480–2.100	18	1
2006	2.100–11.200	22	2
2006	11.200–18.890	21	3
2006	18.890–27.180	20	4
2006	27.180–51.800	21	5

## 4 Proposed methodology

**Figure 2:**
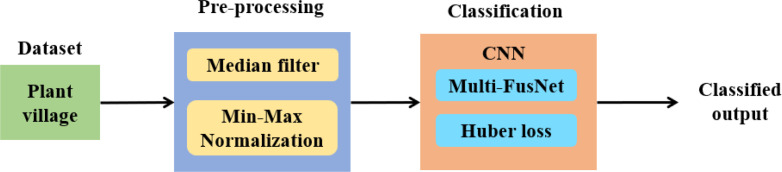
Process of plant leaf disease detection and classification.

The dataset considered in this research is the Plant Village dataset and it is pre-processed using median filter and min–max normalization techniques, which eliminates the noise and normalizes the values in a uniform scale. Subsequently, the Multi-FusNet and improved Huber loss function are incorporated in the CNN model for classifying different classes of plant leaf diseases. **Figure 2** presents the process of plant leaf disease detection and classification.

### 4.1 Pre-processing

Images in the dataset are given an as input to the pre-processing phase, where median filter and min–max normalization techniques are used. These pre-processing techniques are explained as follows.

- Median filter—It is a non-linear processing method utilized for eliminating the noise in imageswhile preserving the edges. This method eliminates the noise caused by shadows, without blurring the edges. This process maintains significant features like texture and shape of disease-affected regions, which are essential for classification.
- Min–max normalization—It is a data scaling method utilized for normalizing pixel intensity values to a fixed range (0,1). Initially, minimum and maximum pixel intensity values are identified in data and employed the min–max normalization formula, which is given in Equation 1,

(1) $$X^{\prime }=\frac{X-X_{min}}{X_{max}-X_{min}}$$

In Equation 1, X represents actual values, $X^{\prime }$ denotes normalized values, $X_{max}$ indicates maximum values of pixel intensity, and $X_{min}$ represents the minimum value of pixel intensity. This process ensures that all pixel values are in the same range, which makes the input uniform for classification. It also prevents features with large scales from dominating otherswhile ensuring that the model concentrates equally on all features.

### 4.2 Classification

CNNs are multiple layered network architectures widely utilized for resolving different image classification issues. The integration of CNN properties like parameter sharing and sparsity connection has effectively minimized the amount of parameterswhile comparing with the conventional deep neural network (DNN) approach. The developed CNN architecture includes the series of convolutional layers, batch normalization, pooling layers, activation function, fully connected, and softmax layers. The detailed description of these layers is explained in sections below.

#### 4.2.1 Convolution layer

It is the initial layer of neural network and a developing block of architecture. The convolutional layer has numerous feature detectors called filters or kernels, which slide through strides across a whole image for identifying the existence of a specific feature. The developed architecture utilizes three convolution layers, namely, conv-1 with 16 kernels, conv-2 with 32 kernels, and conv-3 with 64 kernels.

#### 4.2.2 Batch normalization layer

It is an approach to train the neural network that standardizes the input to the layer for every mini-batch. As a result, the learning process trains and stabilizes the neural network with a requirement of less amount of training epochs. Consider the mini-batch $B=\{x_{1-m}\}$. The components $x_{i}$ are normalized by measuring the mini-batch mean $\mu_{B}$ and the variance represented by $\sigma_{B}^{2}$. The mathematical formula for normalized activation is measured by using Equation 2,

(2) $$\hat{x}=\frac{x_{i}-\mu_{B}}{\sqrt{\sigma_{B}^{2}+\epsilon_{B}}}$$

In Equation 2, $\epsilon_{B}$ is incorporated to the variance of the mini-batch in numerical stability. The default value for $\epsilon_{B}$ is 10^−5^. The process of batch normalization shifts and scales the activation by utilizing the transformation given in Equation 3,

(3) $$y_{i}=\gamma_{B}{\hat{x}}_{i}+\beta_{B}$$

In Equation 3, $\gamma_{B}$ and $\beta_{B}$ represent trainable parameters. The developed architecture utilizes three batch normalization layers with 32, 64, and 128 parameters, respectively.

#### 4.2.3 Max pooling layer

The primary function of the pooling layer is to minimize the output dimension of a provided convolution layer, which reduces the executional complexity in the next layer. The max pooling layer is the most popular method utilized for pooling that separates the image to sub-area rectangles determined through kernel and returns the highest value of every kernel. The developed architecture utilizes the max-pooling process with a 2×2 kernel size and a stride of 2.

#### 4.2.4 Activation function layer

The activation function layer follows the pooling layer. This layer implements non-linearity to the neural network. By utilizing non-linearity, the generated result of the past layer is changed. It is utilized for restricting the generated results. The mathematical formula for the rectified linear unit (ReLU) activation function is given in Equations 4 and 5,

(4) g(z)=max(0,z)

(5) $$\frac{d}{dz}g\left(z\right)=\{\begin{array}{c} 1,\;\;\;\;\;\;\;if\;z>0\\ 0,\;\;\;otherwise \end{array}\;$$

The activation function minimized the issue of the vanishing gradient. ReLU generates a sparser representation, because zero is a gradient outcome in full zero.

#### 4.2.5 Fully connected layer

It is structured similarly to the organized arrangement of neurons in conventional neural networks. Consequently, every neuron in a fully linked layer is straightly coupled to each neuron on past and subsequent layers. The primary disadvantage of a fully connected layer is that it has several parameters, which need extended execution in training processes. Therefore, the developed method has one fully connected layer that has been utilized. Finally, the result of the neural network is fed to the softmax activation function, which turned the real-value result of the fully connected layer into a possibility distribution.

### 4.3 Multi-FusNet–convolutional neural network

**Figure 3:**
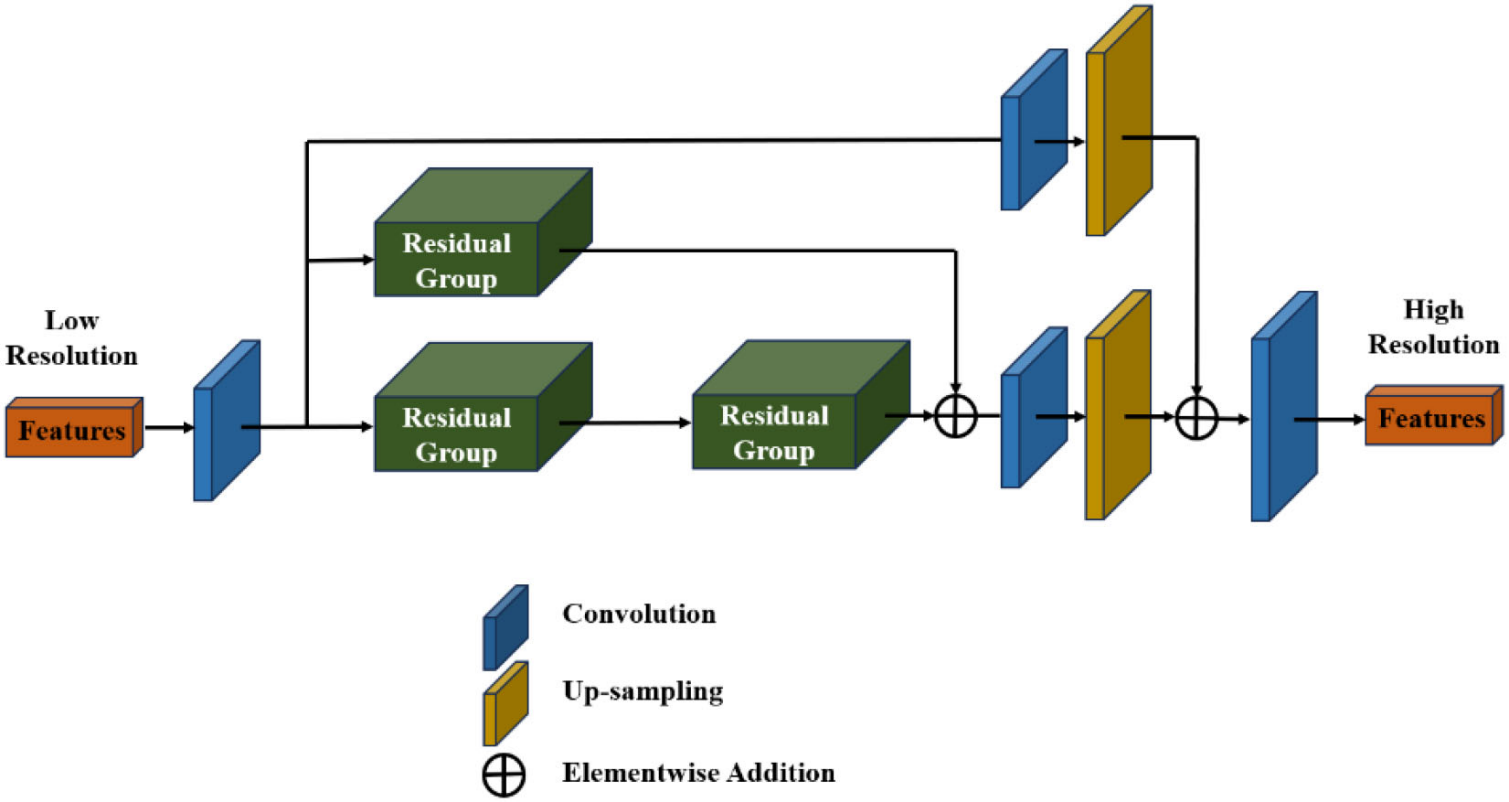
Architecture of Multi-FusNet–CNN.

The developed network is created based on multipath residual architecture, which gives a broader network instead of a deeper one, resulting in effective and faster execution. The network called Multi-FusNet–CNN includes four primary modules, namely, feature extraction, residual group (RG) enlargement, low-level fusion, and Huber loss function. The RG architecture has been developed by combining the residual channel attention network (RCAN) with multiple identical residual links. Because of the increased diversity in developed architecture, data flowamong RG blocks gradually rises, which helps minimize executional complexity. The developed cascading topology in the Multi-FusNet–CNN includes three various paths that develop into multipath residual configuration. **Figure 3** presents the architecture of Multi-FusNet–CNN.

Every RG block includes stacked residual channel attention (RCA) and short residual skip connection in a block. The initial block includes two stacked RG blocks; the second path contains one RG block cascaded with the initial path; the third path bypasses low-level data of the initial layer and fuses it with the high-level features of RG. For processing the image enlargement, the pixel shuffle method is used to transmit low-level feature maps to various channels and shuffles features to enlarge them.

The RCAN method provides high accuracy; the large number of parameter outcomesg iven given less execution time makes its implementation in real time difficult. To develop the lightweight architecture, a certain quantity of RGs is minimized and the multiple path residual network is included in the method. The design enhances the accuracy and execution speed, resulting in a much effective method compared with the utilization of a non-cascading architecture. To enhance the method’s capability for extracting shared attribute data, it leverages low-level features from initial CNN layers and shared high-level features from the multipath residual network. Several DL algorithms suffered from over-smooth degradation because of the limited high-frequency data in the CNN layer, causing bad images on a large scale. By including the pixel shuffling fusion method, these drawbacks are mitigated and offered high-quality outcomes.

#### 4.3.1 Residual group

The RG block is a feature extraction block with less resolution. The developed RG is created by employing an identical residual connection at the edge of N, which is a sequence of statistical channel attention networks, where N>0. The result of RG is represented through $O_{RG}$, and its mathematical expression is given in Equation 6,

(6) $$O_{RG}=I_{RG}+W_{RG}\left(F_{RCA_{N}}\right)$$

In Equation 6, $I_{RG}$ is the input of the RG block, $W_{RG}$ is a weight parameter in the RG block, and $F_{RCA_{N}}$ represents the channel-wise feature of the RCA block. The mathematical formula of the channel-wise feature from RCA is provided in Equations 7 and 8,

(7) $$F_{RCA_{N}}=H_{RCA}^{N}\left(F_{RCA_{N-1}}\right)$$

(8) $$F_{RCA_{0}}=H_{RCA}^{0}\left(I_{RCA}\right)$$

In the above equations, $F_{RCA_{N}}$ and $F_{RCA_{N-1}}$ represent the results of *N*th and (*N−*1)th RCA. $H_{RCA}$ represents the respective process function of RCA. $F_{RCA_{0}}$ denotes the result of the initial RCA block, and $I_{RG}$ indicates the input of an initial RCA block. The channel attention (CA) mechanism utilizes interdependencies between feature channels. The CA method focuses more on informative features and enhances the ability of the model.

#### 4.3.2 Multiple path residual

The integration of RG blocks in the multiple path residual architecture is utilized in the developed method. Based on multiple path residual signals, the broader residual network effectively enhances the execution speed and accuracy of the method compared to the deep residual model. These enhancements are relevant in maximizing the multiplicity of a broad residual model. The multiplicity suggests the number of probable routes from the input to the output layer. The sequence of two RG blocks is used in an initial path of the developed method, and one RG block is assigned in the second path. The mathematical formula for a multipath result of the developed method is given in Equation 9,

(9) $$O_{MR}=O_{RG}^{2}+O_{RG}^{1}$$

In Equation 9, $O_{RG}^{2}$ and $O_{RG}^{1}$ represent the results of two RG blocks in the initial path and RG in second path, respectively. $O_{MR}$ denotes the result of the developed multipath residual network.

#### 4.3.3 Pixel shuffle fusion

The low-level feature sharing technique is used for improving the sharpness of reconstructed outcomes. Because of the low-level features of the initial layer that include much high-frequency data, sharing enhances the challenging weakness of CL algorithms in recovering sharp attributes of edges and lines. At the same time, it protects the developed method against the degradation of over-smoothing. The method uses a pixel shuffle fusion technique for bypassing low-frequency features of initial layers of the developed network to high-level features. The developed method assigns a pixel shuffle for up-sampling images. Based on the feature sharing technique, features of the initial layer, which are high-level through the pixel shuffle technique, are fused with up-sampling attributes of the multiple path residual network. The mathematical formula is given in Equation 10,

(10) $$PS_{Fus}=PS_{1}\left(W_{MR}\left(O_{MR}\right)\right)+PS_{2}\left(W_{p}\left(F_{L}\right)\right)$$

In Equation 10, $PS_{1}$ and $PS_{2}$ denote pixel shuffle up-sampling in a multiple path residual model and low-level feature sharing, respectively. $W_{MR}$ and $W_{p}$ indicate a convolution process of multiple path residual results and low-level features, respectively. The mathematical formula for pixel shuffle is given in Equation 11,

(11) PS(U)i,j,c=U[ia][ja], C·a·mod(i,a)+C·a·mod(i,a)+c

In Equation 11, PS(U)  represents the result, a denotes the scale factor, i, j represent the pixel coordinates, and c indicates channel location. The mathematical formula is given in Equation 12,

(12) $$SR=W_{SR}\left(PS_{Fus}\right)$$

In Equation 12, $PS_{Fus}$ represents the result of the fusing pixel shuffle method and $W_{SR}$ denotes the final convolution process to provide the outcome. The developed fusion technique enhances the method’s ability to recover sharp features and enhances the perceptual quality of outcomeswhile mitigating the issue of over-smoothing. The mathematical formula is provided in Equation 13,

(13) $$L_{1}\left(\phi \right)=\frac{1}{n\times m}\sum_{i=1}^{n}\sum_{j=1}^{m}∥SR\left(i,j\right)-y\left(i,j\right)∥$$

In Equation 13, SR and y represent output and reference image, respectively, and n and m denote vectors relevant to training data.

#### 4.3.4 Huber loss function

It is a loss function that is less sensitive to outliers in a data compared to the mean squared error (MSE) utilized in least squares. It gives many stable and reliable outcomes. The process involves the integration of MSE for small errors and mean absolute error (MAE) for huge errors. The mathematical formula for Huber loss function is given in Equations 14 and 15,

(14) $$L\left(x\right)=0.5\times {\left(error\right)}^{2},\;if\;|error|\leq \delta$$

(15) L(x)=δ×|error|−0.5×(δ), if |error|>δ

where error is the variance between the predicted and original value and δ represents the parameter control of transition between two regimes like linear and quadratic regions of the loss function.

#### 4.3.5 Improved Huber loss function

To enhance the outcomes, an improved version of Huber loss function is developed, which is less sensitive to the choice of delta parameter and more effective against outliers and has good optimization properties. Improved Huber loss has a smooth transitionamong linear and quadratic phases of the loss function, where the conventional Huber loss has a sharp transition. The mathematical formula for improved Huber loss is provided in Equations 16 and 17,

(16) $$L_{1}\left(x\right)=0.5\times {\left(error_{1}\right)}^{2},\;if\;|error_{1}|\leq \delta$$

(17) $$L_{1}\left(x\right)=\delta \times |error_{1}|-0.5\times \left(\delta^{2}\right),\;if|error_{1}|>\delta$$

In the above equations, $error_{1}$ represents the varianceamong predicted and original value and δ denotes a hyperparameter that controls the transition from a quadratic to a linear phase of loss function. The δ parameter in improved Huber loss is set to δ=1.0 in all experiments. This value balances between sensitivity to small prediction errors and robustness to outliers, which is essential for handling illumination variations and background noise in images. Improved Huber loss function gives good outcomes for lower loss and higher accuracy than the conventional Huber loss function. This loss function has good optimization properties, with reduced error for oscillation and a smooth transitionamong quadratic and linear phases, making it less sensitive to outliers.

### 4.4 Research implication

The development of Multi-FusNet–CNN with improved Huber loss function has essential implications for both agricultural practices and the broader field of DL-based image classification. In the agriculture field, this article offers scalable, effective, and highly precise solution for plant disease detection, enabling farmers to take timely interventions, minimize crop losses, and enhance the overall yield quality. By developing a method that is computationally lightweight yet robust against outliers, this research makes advanced artificial intelligence (AI) algorithms more accessible for resource-constrained environments, which promotes the adoption of smart farming technologies in growing areas. From a technical perspective, the introduction of multipath residual networks, pixel shuffle fusion, and improved Huber loss function contributes to the advancement of DL-based architectures by addressing general challenges like overfitting, high model complexity, and sensitivity to noisy data.

## 5 Experimental results

The introduced Multi-FusNet–CNN with improved Huber loss algorithm is simulated with the Python 3.7 environment and configurations such as RAM 8 GB, an i5 processor, and Windows 10 (64 bit). The performance metrics considered to evaluate the performance of an introduced Multi-FusNet–CNN with Huber loss algorithm are accuracy, F1-score, precision, recall, and specificity.

**Table 2** presents the performance of improved Huber loss with different performance metrics for the Plant Village and RoCoLe datasets. The standard loss functions considered to evaluate the performance of the introduced improved Huber loss function are exponential loss, cross-entropy loss, focal loss, and the standard Huber loss function. The developed improved Huber loss function obtained 99.95% accuracy, 99.13% F1-score, 99.87% recall, 99.27% precision, and 99.93% specificity on the Plant Village dataset. The developed improved Huber loss function obtained 93.23% accuracy, 92.91% F1-score, 92.86% recall, 93.04% precision, and 93.76% specificity on the RoCoLe dataset.

**Table 2:** Performance of improved Huber loss function.

Methods	Accuracy (%)	Precision (%)	Recall (%)	F1-score (%)	Specificity (%)
Plant village dataset					
Exponential loss	97.37	97.05	96.88	96.21	97.19
Cross-entropy loss	98.09	97.84	97.27	96.79	97.56
Focal loss	98.68	98.23	97.85	97.32	98.14
Huber loss	99.21	98.87	98.33	98.01	98.78
Improved Huber loss	99.95	99.27	98.87	99.13	99.93
RoCoLe dataset					
Exponential loss	91.68	91.55	91.32	91.10	90.89
Cross-entropy loss	92.03	91.86	91.62	91.38	91.07
Focal loss	92.45	92.15	92.04	91.86	91.52
Huber loss	92.87	92.58	92.31	92.17	91.87
Improved Huber loss	93.23	93.04	92.86	92.91	93.76

**Table 3** presents the performance of Multi-FusNet–CNN with different performance metrics. The standard classifiers considered to evaluate the performance of a developed Multi-FusNet–CNN with improved Huber loss function are ResNet, ANN, multi-layer perceptron (MLP), and conventional CNN. The developed Multi-FusNet–CNN with improved Huber loss function obtained 99.95% accuracy, 99.13% F1-score, 99.87% recall, 99.27% precision, and 99.93% specificity when compared to other classifiers. The developed Multi-FusNet–CNN with improved Huber loss function obtained 93.23% accuracy, 92.91% F1-score, 92.86% recall, 93.04% precision, and 93.76% specificity on the RoCoLe dataset.

**Table 3:** Performance of Multi-FusNet–CNN with improved Huber loss function.

Methods	Accuracy (%)	Precision (%)	Recall (%)	F1-score (%)	Specificity (%)
Plant village dataset					
ResNet	98.45	98.17	97.52	97.02	97.84
ANN	98.82	98.52	97.84	97.42	98.34
MLP	99.06	98.83	98.13	97.89	98.79
CNN	99.46	99.03	98.56	98.27	99.13
Multi-FusNet–CNN	99.95	99.27	98.87	99.13	99.93
RoCoLe dataset					
ResNet	91.33	91.10	90.87	90.53	91.03
ANN	91.64	91.47	91.27	91.04	91.44
MLP	92.19	92.02	91.89	91.64	91.89
CNN	92.67	92.43	92.13	92.02	93.21
Multi-FusNet–CNN	93.23	93.04	92.86	92.91	93.76

**Table 4** presents the computational analysis of the proposed Multi-FusNet–CNN model with existing algorithms on Plant Village and RoCoLe datasets. The performance metrics such as training time, inference time, overall execution time, FLOPs, and number of parameters are used to evaluate the performance of the proposed model. When training time increases for the RoCoLe dataset because of higher image variability and real-field complexity, the proposed model consistently maintains lower parameters and FLOPs across both datasets. Moreover, stable and lower inference latency shows that the proposed model is computationally efficient and lightweight for resource-constrained agricultural data, thereby obtaining efficient trade-off between model complexity and classification performance.

**Table 4:** Computational analysis of the proposed Multi-FusNet–CNN model with FLOPs and parameters.

Methods	Training time (s)	Inference time per image (ms)	Overall execution time (s)	FLOPs (GFLOPs)	Parameters (M)
Plant village dataset					
ResNet	35.2	5.1	382	0.72	60.0
ANN	88.4	17.9	821	15.5	138.0
MLP	51.7	7.4	584	4.1	25.6
CNN	58.6	8.7	612	2.9	8.0
**Proposed Multi-FusNet–CNN**	**27.6**	**2.1**	**301**	**0.21**	**3.1**
RoCoLe dataset					
ResNet	42.8	5.6	469	0.72	60.0
ANN	101.3	19.4	912	15.5	138.0
MLP	63.2	8.3	698	4.1	25.6
CNN	69.5	9.6	731	2.9	8.0
**Proposed Multi-FusNet–CNN**	**33.9**	**2.4**	**356**	**0.21**	**3.1**

Bold values represent the proposed model values.

**Table 5** presents an ablation study to show the contribution of every architectural component in a proposed model across the Plant Village and RoCoLe datasets. The results show consistent improvement when RGs and multi-path residual connections are introduced,while showing its significance in improving feature reuse and gradient flow. The incorporation of pixel shuffle fusion shows its effectiveness in the fusion of low-level spatial details with high-level semantic features, which is important for extracting fine disease patterns. By using improved Huber loss, it is determined that loss optimization by itself is insufficient without architectural improvements. The Multi-FusNet–CNN integrates multipath residual learning, pixel shuffle fusion, and improved Huber loss, obtaining a higher performance across both datasets.

**Table 5:** Ablation study of the proposed Multi-FusNet–CNN model across both datasets.

Models	Accuracy (%)	Precision (%)	Recall (%)	F1-score (%)	Specificity (%)
Plant village dataset					
CNN	96.40 ± 0.31	96.29 ± 0.34	96.03 ± 0.36	96.17 ± 0.33	96.34 ± 0.29
CNN + Residual Group	97.35 ± 0.27	97.24 ± 0.29	97.08 ± 0.31	97.14 ± 0.28	97.30 ± 0.26
CNN + Multi Path Residual	97.44 ± 0.25	97.32 ± 0.27	97.20 ± 0.29	97.26 ± 0.26	97.39 ± 0.24
CNN + Pixel Shuffle Fusion	97.83 ± 0.22	97.72 ± 0.24	97.65 ± 0.26	97.68 ± 0.23	97.76 ± 0.21
CNN + Improved Huber loss function	96.59 ± 0.33	96.41 ± 0.35	96.28 ± 0.37	96.37 ± 0.34	96.64 ± 0.31
Proposed Multi-FusNet–CNN	99.95 ± 0.02	99.27 ± 0.05	98.87 ± 0.07	99.13 ± 0.04	99.93 ± 0.02
RoCoLe dataset					
CNN	91.34 ± 0.48	91.25 ± 0.50	91.18 ± 0.52	91.20 ± 0.49	91.30 ± 0.46
CNN + Residual Group	92.05 ± 0.44	91.87 ± 0.46	91.63 ± 0.48	91.74 ± 0.45	91.86 ± 0.43
CNN + Multi Path Residual	92.42 ± 0.41	92.31 ± 0.43	92.16 ± 0.45	92.24 ± 0.42	92.34 ± 0.40
CNN + Pixel Shuffle Fusion	92.75 ± 0.38	92.58 ± 0.40	92.37 ± 0.42	92.45 ± 0.39	92.64 ± 0.37
CNN + Improved Huber loss function	91.45 ± 0.47	91.34 ± 0.49	91.25 ± 0.51	91.29 ± 0.48	91.36 ± 0.45
Proposed Multi-FusNet–CNN	93.23 ± 0.31	93.04 ± 0.33	92.86 ± 0.35	92.91 ± 0.32	93.76 ± 0.28

### 5.1 Analysis of the plant village dataset

**Figure 4:**
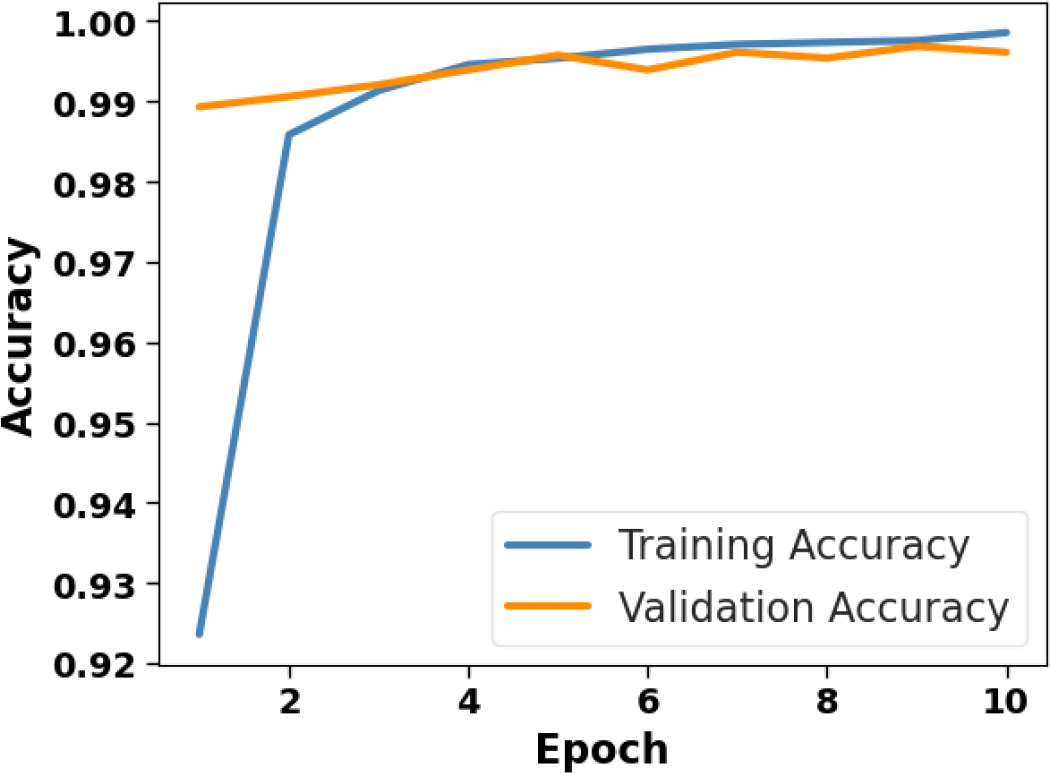
Accuracy *vs*. epoch on the plant village dataset.

**Figure 5:**
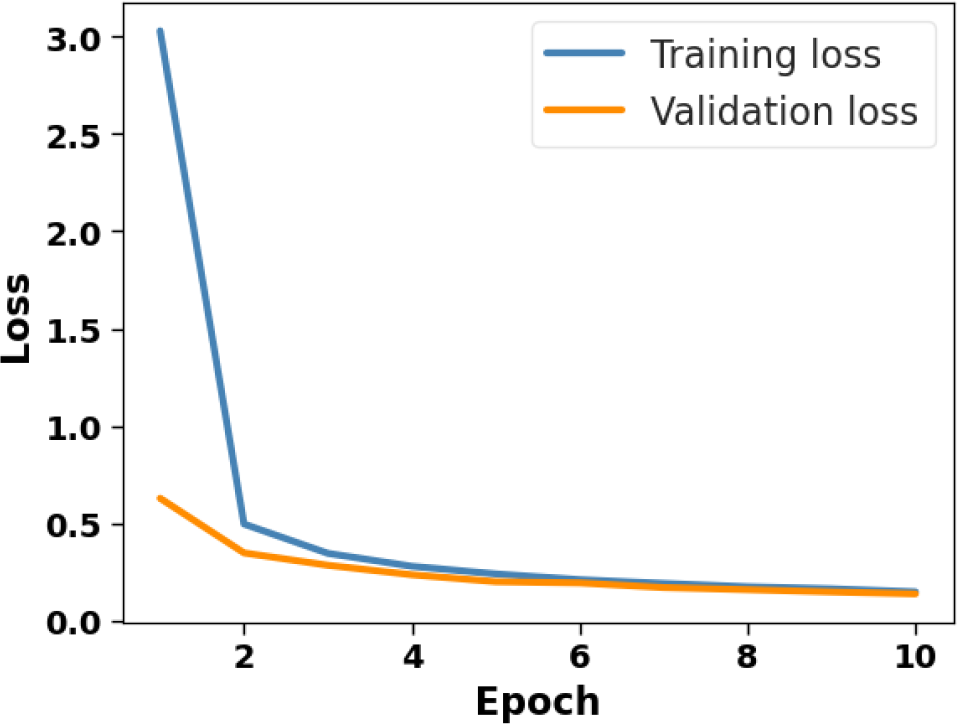
Loss *vs*. epoch on the plant village dataset.

**Figure 6:**
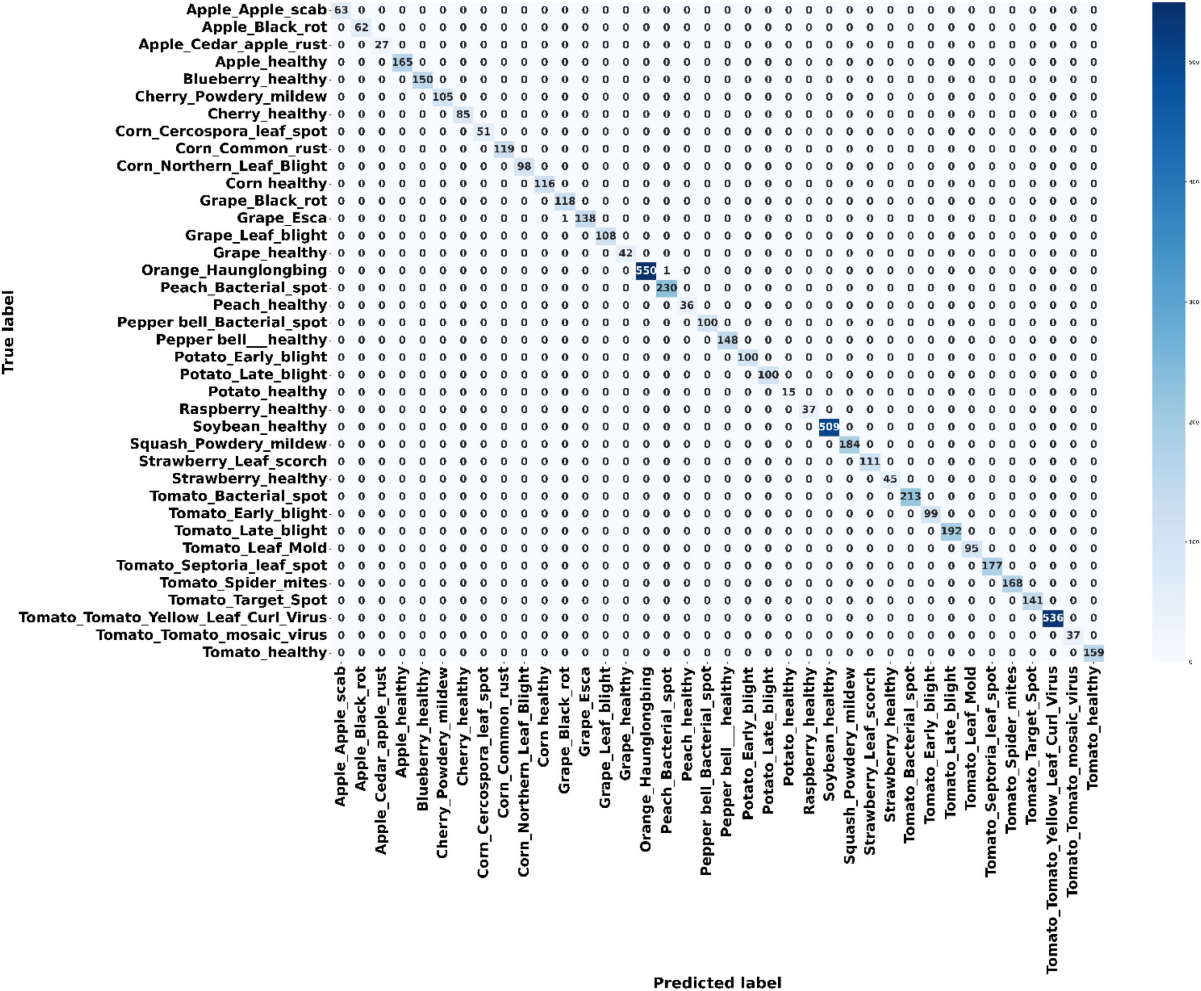
Confusion matrix for the plant village dataset.

**Figure 7:**
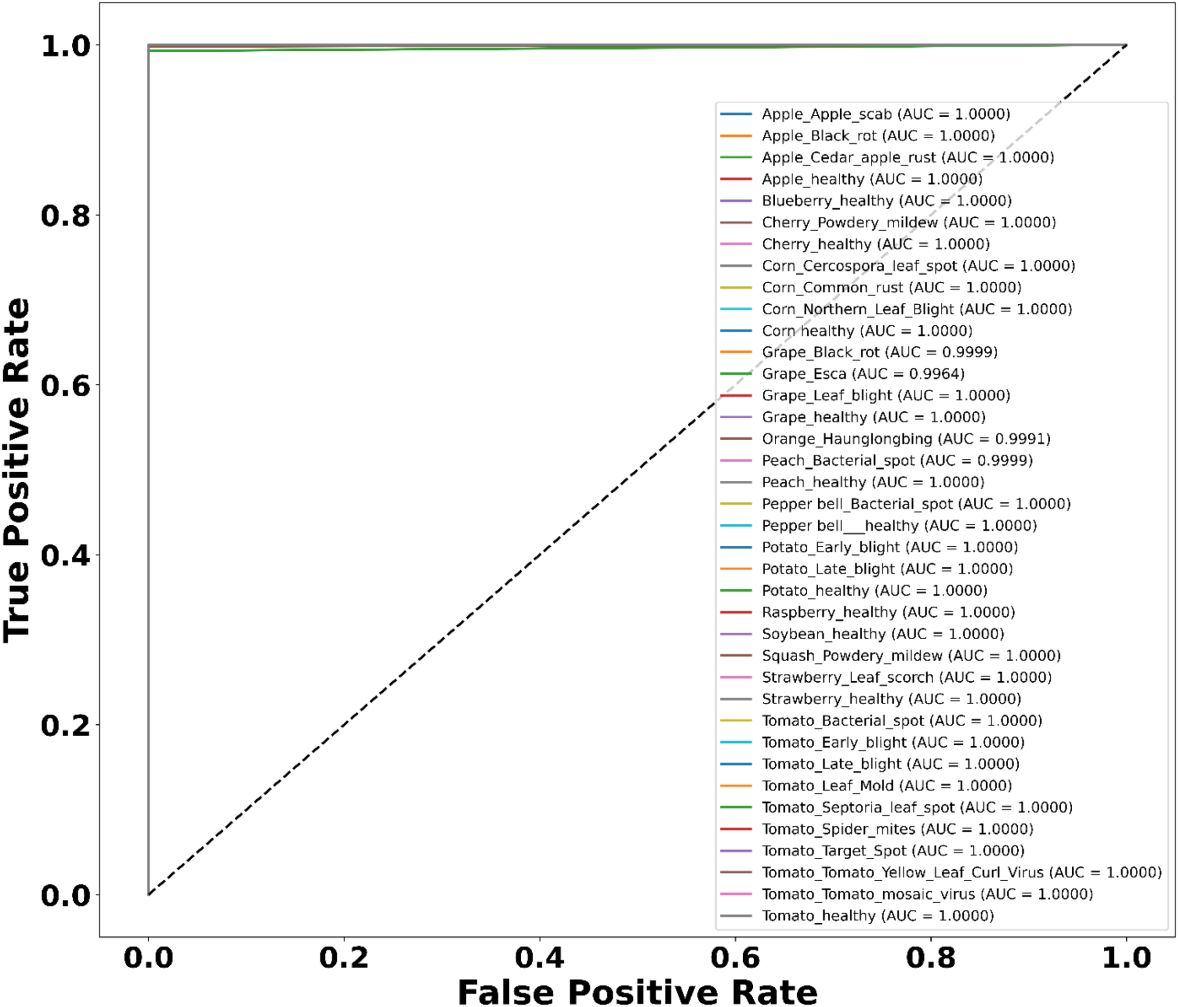
ROC curve on the plant village dataset.

**Figure 4** presents the accuracy *vs*. epoch graph of the Plant Village dataset. **Figure 5** shows the loss *vs*. epoch graph of Plant Village dataset. **Figure 6** presents the confusion matrix of the Plant Village dataset. **Figure 7** shows the ROC curve graph of the Plant Village dataset.

### 5.2 Analysis of the RoCoLe dataset

**Figure 8:**
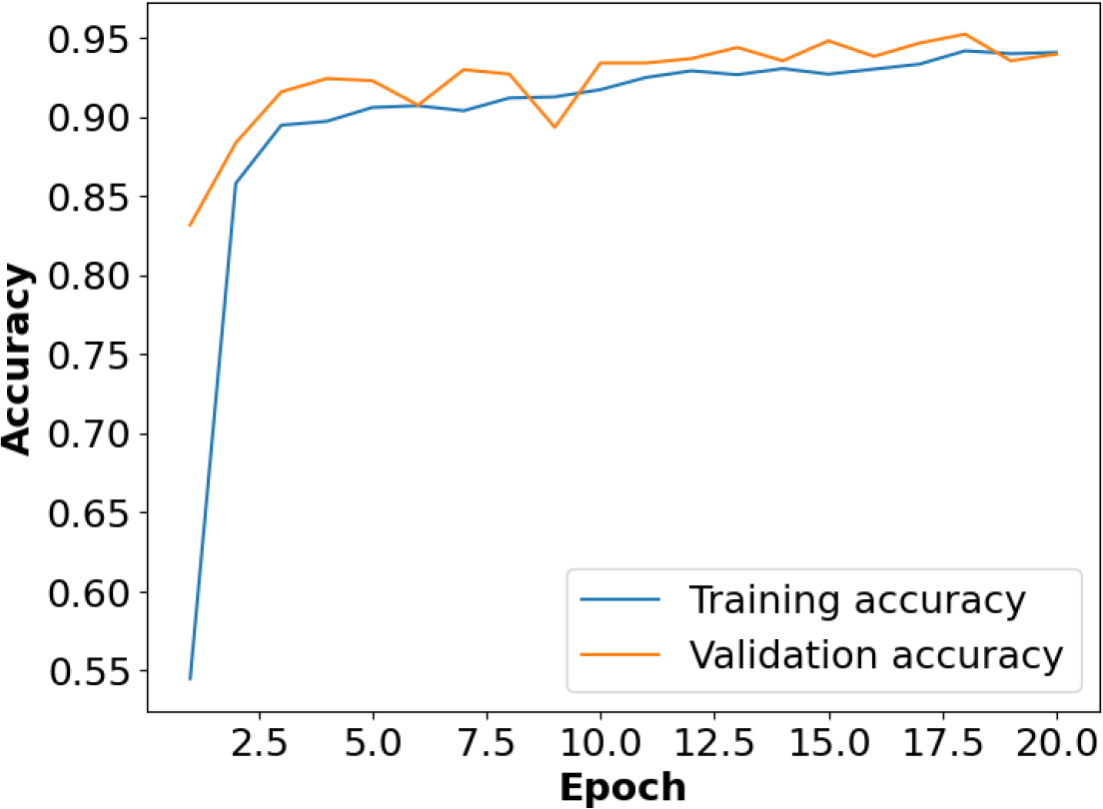
Accuracy *vs*. epoch on the RoCoLe dataset.

**Figure 9:**
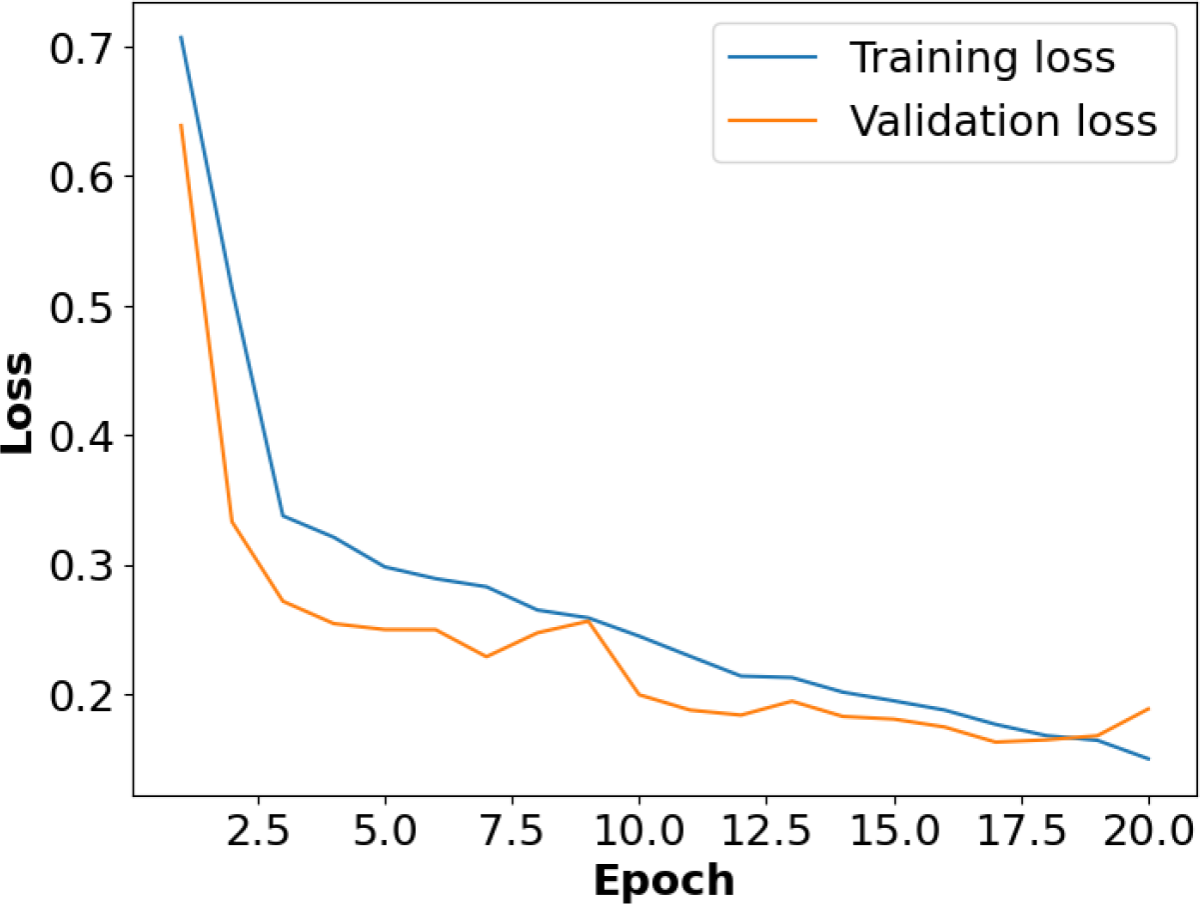
Loss *vs*. epoch on the RoCoLe dataset.

**Figure 10:**
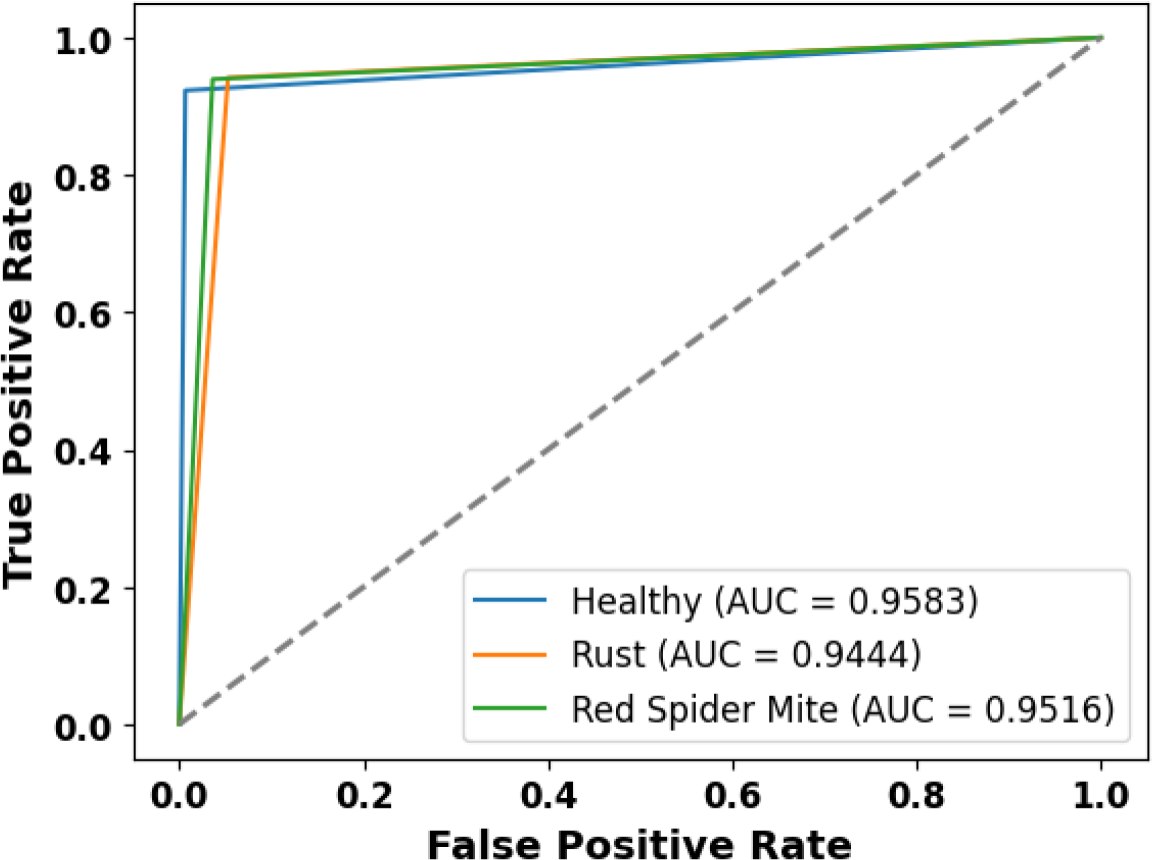
ROC curve on the RoCoLe dataset.

**Figure 11:**
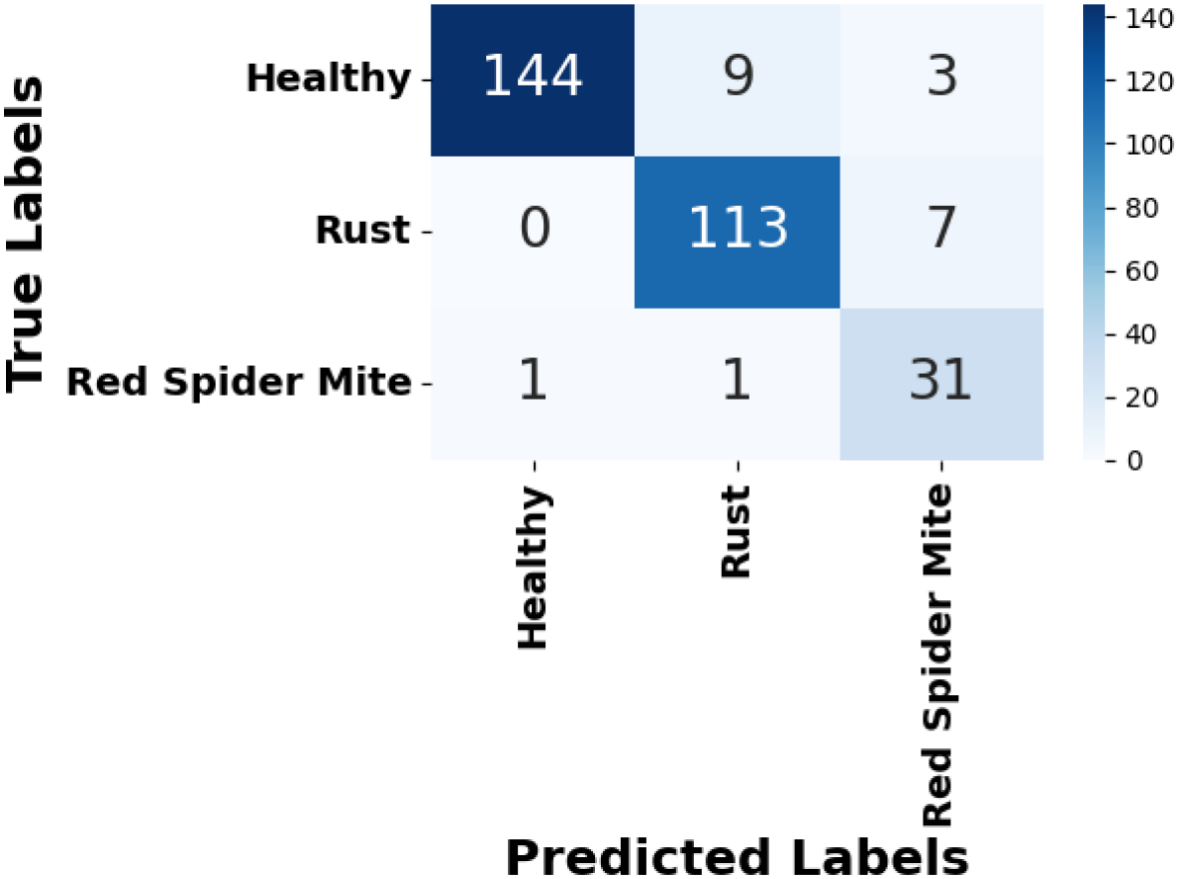
Confusion matrix on the RoCoLe dataset.

**Figure 8** presents the accuracy *vs*. epoch graph for the RoCoLe dataset. **Figure 9** shows the loss *vs*. epoch graph for the RoCoLe dataset. **Figure 10** presents the confusion matrix of the RoCoLe dataset. **Figure 11** shows the ROC curve graph of the RoCoLe dataset.

### 5.3 Comparative analysis

The performance of the developed Multi-FusNet–CNN with improved Huber loss function is compared to existing algorithms like AD-Enet (Kotwal et al., 2024), HRF-MCSVM (Sahu and Pandey, 2023), CNN (Hassan and Maji, 2022), PDICNet (Reddy et al., 2023) and GA-CNN (Moussafir et al., 2022) on the Plant Village dataset. Then, the MobileNetV3 + Swin Transformer (Faisal et al., 2023) and Grabcut method and color analysis process (Hasan RI. et al., 2022) methods are compared on the RoCoLe dataset. The developed Multi-FusNet–CNN with an improved Huber loss function obtained 99.95% accuracy, 99.13% F1-score, 99.87% recall, 99.27% precision, and 99.93% specificity compared to existing algorithms. **Table 6** presents a comparative analysis of the developed Multi-FusNet–CNN with an improved Huber loss function. The proposed Multi-FusNet–CNN model obtains a balanced trade-off between high classification accuracy and computational efficacy for plant disease detection. By combining multipath residual learning with channel attention, the proposed model improves discriminative feature extractionwhile maintaining stable gradient flow. The pixel shuffle fusion mechanism efficiently integrates low-level spatial details with high-level semantic representations, overcomes over-smoothing, and enhances robustness under varying illumination and background conditions. Moreover, the improved Huber loss function mitigates outliers and stabilizes optimization, resulting in better generalization across both datasets.

**Table 6:** Comparative analysis of the proposed Multi-FusNet–CNN model with existing models.

Methods	Datasets	Accuracy (%)	Precision (%)	Recall (%)	F1-score (%)	Specificity (%)
AD-Enet (Kotwal et al., 2024)	Plant Village	99.91	99.87	99.81	99.84	99.95
HRF-MCSVM (Sahu and Pandey, 2023)		98.9	NA	NA	97.8	NA
CNN (Hassan and Maji, 2022)		99.73	NA	NA	NA	NA
PDICNet (Reddy et al., 2023)		99.73	99.83	99.72	99.78	NA
GA-CNN (Moussafir et al., 2022)		98.1	NA	NA	NA	NA
Proposed Multi-FusNet–CNN with Huber loss		99.95	99.27	98.87	99.13	99.93
MobileNetV3 + Swin Transformer (Faisal et al., 2023)	RoCoLe	84.29	84.67	84.29	83.64	NA
Grabcut method and color analysis process (Hasan RI. et al., 2022)		90	NA	NA	NA	NA
Proposed Multi-FusNet–CNN with Huber loss		93.23	93.04	92.86	92.91	93.76

### 5.4 Generalization ability

The developed Multi-FusNet–CNN with an improved Huber loss function demonstrates strong generalization ability by efficiently handling variations in plant leaf images like differences in size, shape, color, and environmental conditions. The combination of multipath residual structures and pixel shuffle fusion ensures that low-level and high-level features are captured well, and preserves significant details for classification. The improved Huber loss function further stabilizes training by minimizing the sensitivity to outliers, thereby enabling the method to maintain high accuracy across different datasets. As the outcome, the method avoids overfitting and consistently performs well on unseen data,while proving its robustness and adaptability for real-world agricultural applications.

## 6 Discussion

Environmental variations like non-uniform lighting, shadows, and complex backgrounds affect low-level visual features by distorting color intensity, edge responses, and texture patterns. The high-level features learned in deeper layers encode semantic and structural representations of plant diseases, which are more invariant to such noise. The proposed Multi-FusNet–CNN uses the fusion of low-level and high-level features to balance fine-grained detail preservation with semantic robustness. When low-level features are used for discriminative local features that are crucial for disease detection, high-level features suppress background interference and illumination-related distortions. Though the proposed Multi-FusNet–CNN model obtains high accuracy on the Plant Village dataset, this is captured under laboratory-controlled conditions with uniform backgrounds and lighting. Such conditions increase the risk of overfitting and overestimate real-world performance. To overcome this drawback, the proposed model is evaluated on the RoCoLe dataset that includes field-acquired images with complex backgrounds and varying illumination, where consistent performance enhancements are obtained.

### 6.1 Limitations

The proposed Multi-FusNet–CNN obtains high performance; its robustness has not been validated on a large scale in agricultural datasets with extreme environmental variations. The feature fusion strategy requires adaptation when it is extended to unseen crops or disease classes.

## 7 Conclusion

The detection and classification of plant leaf disease have become challenging because of the overlap between different types of diseases. To mitigate these limitations, this research developed the Multi-FusNet–CNN with an improved Huber loss function to classify multiple classes of plant leaf diseases. Images in the dataset are pre-processed by a median filter and min–max normalization to enhance image quality. Here, Multi-RG with cross-filtering fusion is integrated and the pixel shuffling fusion method is developed for fusing low-level features to up-sampled features. Then, the improved Huber loss function is introduced in the Multi-FusNet–CNN, which is effective for outliers and improves the generalization capability of the model during the training process. The developed Multi-FusNet–CNN with improved Huber loss function obtained 99.95% accuracy, 99.13% F1-score, 99.87% recall, 99.27% precision, and 99.93% specificity when compared with existing conventional techniques.

### 7.1 Future work

In future research, the method is extended to identify multiple diseases that occur simultaneously on a single leaf using multi-label classification techniques. Additionally, attention mechanisms or transformer models are combined to further improve feature extraction. The expansion of model testing to real-time field data also validates its robustness under uncontrolled environmental conditions.

## Data Availability

The original contributions presented in the study are included in the article/supplementary material. Further inquiries can be directed to the corresponding author.
